# Two decades on - cardiothoracic surgical care practitioners in the UK: a narrative review

**DOI:** 10.1186/s13019-020-1089-2

**Published:** 2020-02-22

**Authors:** Mohammed Bahran Shegafi, Samer Nashef, Roksolana Starodub, Gerry Lee

**Affiliations:** 1 Kings College London, Florence Nightingale Faculty of Nursing, Midwifery & Palliative Care, James Clerk Maxwell Building, 57 Waterloo Road, London, SE1 8WA UK; 2 King Abdullah Medical city, Makkah, Saudi Arabia; 3 Papworth Hospital, Papworth Road, Cambridge Biomedical Campus, Cambridge, CB2 0AY UK

**Keywords:** Surgical care practitioner, Cardiothoracic surgery, Review, Clinical outcomes

## Abstract

**Background:**

The role of Surgical Care Practitioner (SCP) was first introduced by the NHS in the field of cardiothoracic surgery more than two decades ago to overcome the chronic shortage of junior doctors, and subsequently evolved into other surgical specialties. This review aims to provide evidence on the current situation of SCPs’ clinical outcomes within their surgical extended role, with an emphasis on the cardiothoracic surgical field.

**Method:**

A systematic search of PubMed, Scopus, Embase via Ovid, Web of Science and TRIP was conducted with no time restriction to explore the evidence on SCPs. All included articles were reviewed by three researchers using the selection criteria, and a narrative synthesis was undertaken.

**Findings:**

Ten out of the 38 studies identified were selected for inclusion. Only one study specifically investigated cardiothoracic SCPs. Three themes were identified: (1) clinical outcomes (six studies), (2) workforce impact (two studies) and (3) colleagues’ opinions (two studies). All studies demonstrated that SCPs provided safe practice, added value and were of benefit to workforce environments and surgical teams.

**Conclusion:**

Although the current literature provides assurances that the presence of SCPs within surgical teams is beneficial in terms of their clinical outcomes, their impact on the workforce and colleagues’ opinions, a significant gap was identified around the SCPs’ role within their surgical extended role, specifically in cardiac surgery. Thus, prospective clinical research is required to evaluate SCPs’ clinical impact.

## Background

Across the UK, the National Health Service (NHS) has introduced new professional roles within multi-disciplinary teams due to the chronic shortage of doctors and the implementation of the European working time directive [1]. These professions are known as Medical Associate Professions and include Physician Associates (PA), Physicians’ Assistants (anaesthesia) [PA(A)], Advanced Critical Care Practitioners (ACCP) and Surgical Care Practitioners (SCP) [2, 3]. This workforce transformation was introduced to overcome the shortage of doctors, ensuring that the NHS’s high quality care remains accessible to everyone and maintaining its ranking as a leading provider of high-quality healthcare within the current climate [4].

An SCP is defined by the Royal College of Surgeons as:

“… *a non-medical practitioner, working in clinical practice as a member of the extended surgical team, who performs surgical intervention, pre-operative and post-operative care under the direction and supervision of a consultant surgeon*.” [5]

SCPs were first introduced in the UK in the cardiothoracic surgical setting in the early 1990s in Oxford, and the role then evolved into other surgical specialities, including orthopaedics and general surgery [6]. According to the recent workforce report published by the Society for Cardiothoracic Surgery in Great Britain and Ireland, the number of SCPs is expected to increase within the cardiothoracic field [7]. Despite over 20 years of SCPs in cardiac surgery in the UK, the initial literature search revealed a lack of research evidence surrounding their role in this setting. However, even within other surgical specialities, there is a paucity of evidence-based literature on SCPs’ contribution and impact. This paper aims to provide evidence on the current situation by systematically searching, reviewing, appraising and synthesising current evidence on the clinical impact of SCPs.

## Main text

### Search strategy

A narrative literature review was conducted to provide a comprehensive overview that would facilitate an understanding of the clinical outcomes associated with SCPs. An initial scoping search was performed using Google Scholar. A further in-depth search was conducted using PubMed, Scopus, Embase via Ovid, Web of Science and TRIP. All databases were limited to human studies, published in English, and full-text articles. No timeline restriction was imposed on the database searches, as the initial scoping showed that limited relevant evidence was available. The search keywords and synonyms are illustrated in Table 1.

**Table 1:** Search terms used

Keywords	Synonyms
Surgical care practitioner	Surgical Assistants, Surgeon Assistant, Clinician, Midlevel Providers, Nurse Assistant, Non-physician, Non-Medical, healthcare professionals, Personnel, Operators
Impact	Influence, Effect, Value, Contribution
Clinical Outcomes	Successful Rate, Patient Satisfaction, Operative time, Cost Effectiveness, Infection Rate, Morbidity, Mortality, efficiency
Surgery	Operation, Intervention, Procedures, Cardiac surgery, General Surgery, Orthopaedic Surgery, Vascular Surgery, Urology

### Findings

**Fig. 1:**
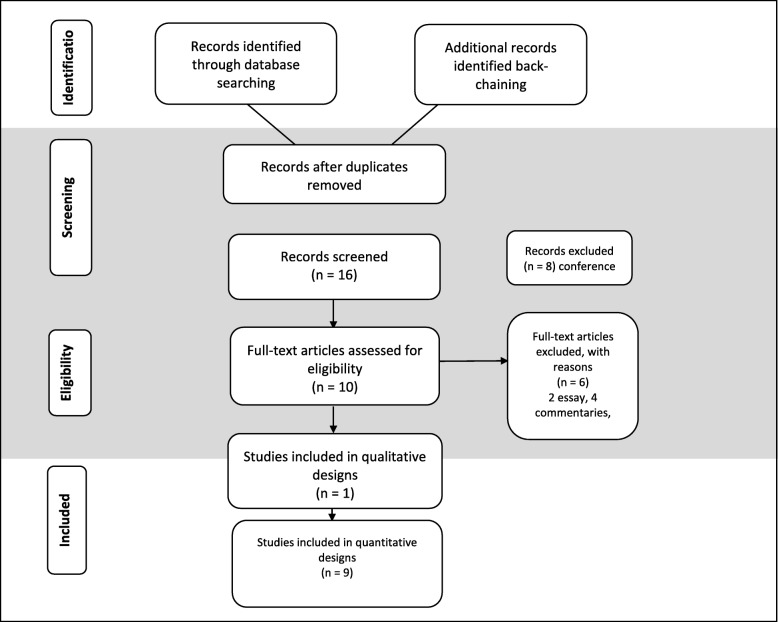
PRISMA Flowchart

A total of 38 articles were identified, with 33 articles sourced from the databases and five additional articles from back-chaining initially being selected for review. All studies were considered eligible except if published as a documentary, essay or review. Twenty-eight articles were subsequently excluded, as detailed in the PRISMA flowchart (Fig. 1).

Three themes were identified from the gathered evidence, namely (1) clinical outcomes [8–13] (2) workforce impact [14, 15] and (3) colleagues’ opinion on the role [16, 17]. The selected studies are summarised in Table 2.

**Table 2:** Studies Summary and Key findings

Author/s	Title	Aims	Design	Outcomes	Sample Size, Sampling Type and Timeframe	Data Collection Method	Key Findings
Alex et al. [8]	Surgical nurse assistants in cardiac surgery: a UK trainee’s perspective	To assess the impact of SCPs on surgical training based on a comparative audit of case-mix and outcome of coronary revascularizations assisted by SCPs assistants vs. surgical trainees	Prospective Audit	Assisting in surgery, harvesting saphenous veins and radial arteries as bypass conduits	1300 patients, convenience sample over two years	Data were entered into the Patient Analysis and Tracking System (PATS) database	SCPs were efficient in assisting surgeons compared to surgical trainees: Operation time (P = 0.0001), Cross-clamp time (*P* = 0.0001)
Martin et al. [9]	The surgical care practitioner: a feasible alternative. Results of a prospective 4-year audit	To audit the volume and outcomes related to the SCP service	Prospective Audit	Safety in performing minor surgical procedures, i.e., removal of sebaceous cysts, skin tags, basal cell papillomas and lipomas	381 patients, convenience sample over four years	All prospectively collected data regarding SCP-managed patients were retrospectively audited.	“SCP is feasible and safe, contributes positively to waiting times and is acceptable to patients”.
Hickey and Cooper [10]	Varicose vein surgery performed by a surgical care practitioner	To assess the contribution of SCPs when performing day-case varicose vein surgery	Prospective research with unclear design	Varicose vein operations, performing leg avulsions and groin wound closure.	327 patients, convenience sample over four years.	Not clear	The mean number of legs treated per list during the six months prior to the implementation of the surgical care practitioner role was 3.3, and this number was raised to 4.7 during the six months from February to July 2007. The six-week follow-up showed no differences in outcome between consultant-performed procedures and those carried out by surgical care practitioners.
Palan et al. [11]	The trainer, the trainee and the surgeons’ assistant clinical outcomes following total hip replacement	“To investigate whether there is an association between surgical outcome and the grade of the operating surgeon (trainees vs. trainers), and whether there is any difference in outcome if surgeon’s assistants assist with the operation rather than trainees (surgeons’ assistants vs. trainees).”	Prospective Cohort Study	Assisting in orthopaedic surgeries.	1501 patients, Consecutive over three years	Postal questionnaires and patient records	The mean operating time significantly decreased, from 90 min to 65 min, when the surgeon was assisted by a SCP (*p* < 0.001).
Williams et al. [14]	Telephone clinic follow-up following carpal tunnel decompression	Investigating the feasibility of using telephone clinics in the routine follow-up of patients following carpal tunnel decompression	Prospective Service Evaluation	Conducting Telephone Clinics to follow up patients.	598 patients, convenience sample over two years.	Pre-determined questionnaires but not clear how delivered	The employment of SCPs in the telephone clinic could save in total approximately £45,958.
Kumar et al. [15]	The general surgical care practitioner improves surgical outpatient streamlining and the delivery of elective surgical care	To examine whether the SCPs could reduce the misdirection of outpatient referrals. To assess whether the SCP could manage post-operative follow-ups via telephone with all elective, benign, major UGI laparoscopic surgery patients	Prospective Audit	Assessing all outpatient referrals. Conducting telephone follow up postoperatively	1448 patients, convenience sample over one year	From hospital record management system	The inclusion of the SCP prevented inappropriate referrals of 175 new patients, saving approximately 35 new outpatient appointments per month.
Quick [16]	The role of the surgical care practitioner within the surgical team	To determine whether SCPs bring benefits	Qualitative/ Autoethnography	Assisting and undertaking surgical procedures	Six senior surgeons	Face-to-face interviews	SCPs added benefits to the patient, members of the surgical team, the practitioner and the employing organisation
Quick [12]	Evaluating a specialist nurse’s role in a general paediatric surgical team	To assess the contribution of the SCP when performing operative procedures.	Retrospective Audit	Performing operative procedures, such as circumcisions. Assisting during day-case elective surgery.	147 patients/ 2 years, retrospective convenience sampling	Patient records	SCPs provide an efficient and safe service with zero rate of complications.
Tingle et al. [13]	Performance and learning curve of a surgical care practitioner in completing hip aspirations	To examine the learning curve and competence of the SCP in performing hip aspirations.	Retrospective Service Evolution	Performing hip aspirations surgeries	510 patients/ five years, retrospective convenience sampling	Patient records	SCPs’ failure rate when performing hip aspiration was significantly lower than that of the surgeons (P < 0.001). With advancing SCP experience, the failure rate dropped to 3.5% from 12.4% with the first 210 cases (*P* = 0.006).
Barry [17]	“How can the presence of a surgical care practitioner improve training for staff who are learning how to scrub for robotics cases in a urology theatre?”	To examine the contribution of the SCP in supporting the learning needs of the junior scrub staff in urology operating theatres	Cross-sectional Survey	Assisting in urological robotics surgeries	Eight junior scrub practitioners	Online questionnaire using SurveyMonkey	The presence of the SCPs enhanced the learning of the junior theatre team in urology

### Clinical outcomes

Six studies were included under this theme, of which two were prospective empirical studies [10, 11], three were audits [8, 9, 12], and one was a service evaluation [13]. All but two [9, 12] of the studies’ themes differed in terms of surgical setting. Only one investigated SCPs in cardiac surgery [8], while the others were based in general surgery [9, 12], vascular surgery [10] and orthopaedics [11, 13]. All studies were prospective except for two [12, 13]. As the studies under this theme differed in their surgical settings and nature (i.e., research, audit and service evaluation), the results of each study will be examined separately.

The presence of the SCP as a member of the extended surgical team in cardiac surgery was not only found to be significant in improving clinical outcomes compared to surgical trainees but also was extremely helpful not only to assist but even further to teach junior trainees the technique of conduit harvesting. This result was inferred in an audit based on a case-mix comparative analysis and the outcomes of coronary artery bypass grafts assisted by SCPs compared to surgical trainees [8]. Statistically significant differences were found in operation time (*P* = 0.0001) and cross-clamp time (P = 0.0001) between the study’s groups, favouring the SCPs’ group over the surgical trainees’ group, even though the mean number of grafts was similar between the two groups (*P* = 0.2). Furthermore, SCPs were found to provide an efficient and safe service when employed to perform minor surgical procedures, such as the removal of sebaceous cysts, skin tags, basal cell papillomas and lipomas, based on an audit conducted over four years [9]. Similarly, in a vascular surgical setting, SCPs were found to contribute positively to day-case varicose vein surgeries [10].

Moreover, the results of a multicentre cohort study in an orthopaedic surgical setting revealed that mean operating time significantly decreased – from 90 min to 65 min – when surgeons were assisted by SCPs (*P* < 0.001) [11]. This finding is supported by a multicentre service evaluation [13], which found that SCPs’ failure rate when performing hip aspirations was significantly lower than that of surgeons, 8.6% vs. 20.7%, respectively (*P* < 0.001).

### Workforce impact

Inclusion of SCPs can provide a valuable contribution to district hospitals [14, 15]. In Williams et al.’s [14] study, an SCP who worked within the orthopaedic department was employed to conduct a telephone clinic to follow up patients who had undergone carpal tunnel decompression surgery. Cost analysis revealed that the employment of SCPs in the telephone clinic could save approximately £45,958 over two years. In another study, outpatient clinic activity was improved, as SCPs prevented inappropriate referrals of 175 new patients – approximately 12.0% of the total general surgical outpatient workload – saving an average of 35 new outpatient appointments per month [15].

### Colleagues’ opinion

Only two studies investigated the value of SCPs from the team perspective. Quick [16] assessed surgeons’ opinion, while Barry [17] investigated the opinions of junior scrub nurses. Both agreed that SCPs enhanced patients’ experience, and also provided benefits to members of the surgical team, the practitioner and the employing organisation [16]. SCPs enhanced the learning of the junior theatre team in urology [17].

## Discussion

As apparent from the identified themes, SCPs provided safe practice and were regarded as valuable members of the extended surgical team. However, although the SCPs’ role was first implemented in cardiac surgery in the early 1990s, over a decade passed before Alex et al. [8] conducted the first audit on this role. While clinical audit is paramount in examining clinical effectiveness, it should be conducted against defined standards to find out whether the examined practice meets these standards [18, 19]. In contrast to Martin et al. and Quick [9, 12], Alex et al. [8] did not describe the standards against which they examined SCPs. On the other hand, Tingle et al. [13] did not provide any justification for the inferred highly significant difference in the failure rate (*P* < 0.001) favouring SCPs in performing hip aspirations.

Since four of the studies reviewed [8, 9, 12, 13] were audits or service evaluation studies, the true clinical impact of SCPs in cardiac and general surgeries cannot be measured based on these studies [19]. However, even empirical studies investigating the clinical outcomes of SCPs in vascular surgery [10] and orthopaedics [11] are weakened by limitations that diminish the external validity of their results.

Hickey and Cooper’s [10] interpretation was difficult to follow because an insufficient tabular presentation of the results was provided. Furthermore, the baseline characteristics of the samples were omitted, so it is impossible to determine whether the results are confounded by the patients’ demographic and comorbidity factors. The authors also did not provide details on their measurement tools and whether they were valid and reliable. In addition, it was not clear who had collected the data over the four years and how the follow-up was performed. Finally, Hickey and Cooper [10] did not provide information on their statistical methods and how they inferred their results.

Even in the case of Palan et al. [11], when critically examining this evidence, it becomes apparent that the investigation of the difference between surgeons’ assistants and medical trainees was stated as the primary aim, but when examining the results, this issue was investigated as an aspect of the secondary outcomes. Palan et al. [11] revealed that there was no significant difference between outcomes for patients treated by surgeons’ assistants compared to other groups. However, it is not clear how the effectiveness of the assistant in surgery could affect the Oxford Hip Score postoperatively. Furthermore, this study was funded by a medical company, so even though the researchers declared no conflict of interest, a question of credibility might arise.

Despite the methodological flaws highlighted above [10, 11] and the nature of the audit and service evaluation studies [8, 9, 12, 13], their results, supporting the role of SCPs in providing optimal clinical outcomes, are consistent with the recently published systematic review that investigated the addition value of physicians’ assistants and nurse practitioners to surgical/trauma services in the USA in terms of their clinical outcomes and revealed that their inclusion was safe [20]. Although the role of SCPs in the UK differs from that of nurse practitioners and physicians’ assistants in the USA in terms of their education and scope of practice, they are similar in the sense that they are non-medically qualified individuals working in surgical extended roles.

As shown in the results section, to date, there is no source of empirical evidence to support the results inferred by Williams et al. [14] and Kumar et al. [15] that the impact of SCPs positively contributes to the workforce environment. However, Johal and Dodd’s [20] systematic review examined the impact of physicians’ assistants and nurse practitioners from the perspective of workforce impact and found that their inclusion helps to provide high quality service in a cost-effective manner, and this inference confirms the findings of Williams et al. [14] and Kumar et al. [15].

Both medical and non-medical personnel working with SCPs express positive perceptions about the SCPs’ role, as concluded by Quick [16, 17]. However, there are several weaknesses that might affect the findings of these two studies, including the purposive sample used by Quick [16], as all of the participants were senior doctors: junior or middle grade doctors were not included. According to the Royal College of Surgeons of England (2014), SCPs frequently work at junior or middle grade doctors’ level within the surgical team. In addition, Quick [16] stated that the criteria for the sample were that the respondents have been working with the SCP for at least six months. Thus, junior doctors, who may rotate between specialties every three months, were excluded without providing any justification. Furthermore, the context in which Barry [17] carried out his research is considered as a potential source of bias due to the relationship between the researcher and the participants, as the researcher was a senior theatre nurse in Urology before becoming an SCP. Therefore, the researcher’s previous experience as a scrub nurse could also be a contributory factor.

### Limitations

As evident from this review, only one study exists in the literature that highlights the continuation of the SCP in cardiac surgery, and this study was conducted as an audit [8]. Thus, a significant gap exists in the literature regarding the effectiveness of the SCP in cardiac surgery. However, the true impact of the SCP in other surgical settings remains limited, since the majority of the studies on SCPs are either single-centre studies with limited scope or have methodological flaws.

### Implications

This review has significant implications for the SCP profession in cardiac surgery and other surgical settings, since it is the first review of its kind that has been conducted to determine and highlight the contribution of SCPs. The identified limitations and recommendations should be considered to integrate the research into clinical practice, forming evidence-based practice to shape the SCP profession within the surgical setting. In addition, this review has been led by a cardiothoracic SCP, and this may encourage other SCPs to conduct their own empirical research, as the majority of the studies in this review were conducted by surgeons, with the exception of three [12, 16, 17], which were conducted by SCPs.

## Conclusion

Based on the available evidence, the role of SCPs in cardiac surgery has been found to be effective in acting as first assistants or in teaching basic surgical skills to junior doctors. Even within other surgical settings, the presence of the SCP has been found to be of benefit in terms of their clinical outcomes, impact on the workforce and colleagues’ opinions. However, this conclusion is weakened by several limitations that affect its external validity. Thus, this review advocates for prospective clinical research to examine the impact of SCPs in cardiac surgery and other surgical settings.

## Data Availability

Not applicable.

## Abbreviations

ACCP: Advanced Critical Care Practitioners
NHS: National Health Service
PA: Physician Associates
PA(A): Physicians’ Assistants (Anesthesia)
PRISMA: Preferred Reporting Items for Systematic Reviews and Meta-Analyses
